# Photochemical reductive homologation of hydrogen cyanide using sulfite and ferrocyanide†
†Electronic supplementary information (ESI) available. See DOI: 10.1039/c8cc01499j


**DOI:** 10.1039/c8cc01499j

**Published:** 2018-05-15

**Authors:** Jianfeng Xu, Dougal J. Ritson, Sukrit Ranjan, Zoe R. Todd, Dimitar D. Sasselov, John D. Sutherland

**Affiliations:** a MRC Laboratory of Molecular Biology , Francis Crick Avenue , Cambridge Biomedical Campus , Cambridge , CB2 0QH , UK . Email: johns@mrc-lmb.cam.ac.uk; b Harvard-Smithsonian Center for Astrophysics , 60 Garden Street , Cambridge , MA 02138 , USA

## Abstract

Photoredox cycling during UV irradiation of ferrocyanide ([Fe^II^(CN)_6_]^4–^) in the presence of stoichiometric sulfite (SO_3_^2–^) is shown to be an extremely effective way to drive the reductive homologation of hydrogen cyanide (HCN) to simple sugars and precursors of hydroxy acids and amino acids.

## Abstract 2

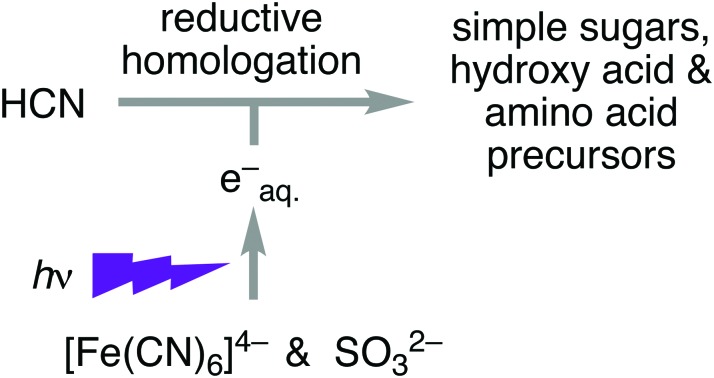
On their own, neither sulfite nor ferrocyanide are efficient sources of photochemically-generated electrons for the reductive homologation of hydrogen cyanide, but together they are.

## 

Our previous, potentially prebiotic, Kiliani–Fischer-like reductive homologation of hydrogen cyanide (HCN **1**) to the simple carbohydrates glycolaldehyde **2** and glyceraldehyde **3**, required the use of either HCN **1** itself, or hydrogen sulfide (H_2_S) as stoichiometric reductants to effect copper(ii) ⇔ copper(i) photoredox cycling (Scheme 1).^1^,^2^ In this chemistry intended to demonstrate ‘protometabolism’,^3^ protons delivered by general acids facilitate direct reduction of nitrile groups by photochemically-generated hydrated electrons. The reaction network is initiated by reduction of HCN **1** to methanimine **4** and hydrolysis of the latter to formaldehyde **5**. Formation of the cyanohydrin of **5**, glycolonitrile **6**, is followed by further reduction and hydrolysis to glycolaldehyde **2**. Another cycle of reductive homologation, *via* glyceronitrile **7**, gives glyceraldehyde **3**. Although prebiotically plausible,^4^ these syntheses are either problematic as regards subsequent use of the sugars in RNA synthesis, or invoke distinct and rather specific geochemical scenarios. Thus, using HCN **1** as the stoichiometric reductant, isocyanic acid **8** (formed upon hydrolysis of cyanogen **9**) traps **2** and **3** in the form of cyclic adducts (Scheme 1).^1^ Using H_2_S as the reductant presents difficulties associated with concentrating such a species in water – its low solubility means that it could most readily be concentrated as its conjugate base, hydrosulfide (HS^–^, p*K*_a_ of H_2_S (∼7)^5^) in alkaline groundwater. Furthermore, the relatively low abundance of copper in Earth's crust would have restricted either chemistry to copper-rich environments, such as those enriched through impact metallogenesis.

**Scheme 1:**
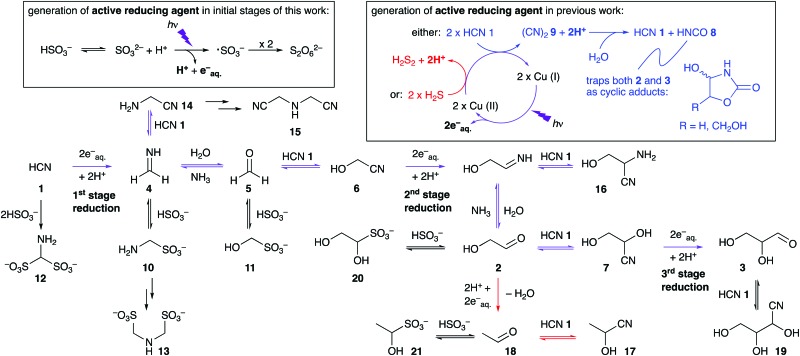
Reductive homologation of HCN **1**. Colour scheme: previously observed chemistry using photoredox cycling of copper(ii) ⇔ copper(i) with the stoichiometric reductant being HCN **1** (blue), or H_2_S (red); previously observed transformations using either reductant (purple); additionally observed new transformations (black).

For reductive homologation of HCN **1** to have been widespread, an alternative to either HCN **1** or H_2_S as reductant would have to have been more globally available, and, if a catalyst was also required, it would ideally be based on a much more abundant metal. Here we describe a potentially widespread prebiotic synthesis of simple sugars and amino acid precursors from HCN **1** using sulfite (SO_3_^2–^, deriving from dissolution of atmospheric SO_2_) as stoichiometric reductant with ferrocyanide ([Fe^II^(CN)_6_]^4–^) promoting the production of hydrated electrons (ESI 1.1†).

We initially explored the photoreduction chemistry of HCN **1** with bisulfite/sulfite alone using direct analysis by ^13^C NMR spectroscopy. After 2.5 h of irradiation, the expected first-stage reduction products of HCN **1**, namely methanimine **4** and its hydrolysis product formaldehyde **5**, were not observed by ^13^C NMR spectroscopy (ESI). Instead, aminomethanesulfonate **10** and hydroxymethanesulfonate **11**, the bisulfite adducts of **4** and **5**, respectively, were observed together with aminomethane-disulfonate **12**^6^ and iminodimethanesulfonate **13** (Scheme 1). The identities of these products were confirmed by comparing their spectral properties with those of authentic compounds (ESI). After a longer irradiation time (5 h), the first-stage Kiliani–Fischer homologation products, glycolonitrile **6**, glycine nitrile **14** and iminodiacetonitrile **15** were observed. Most importantly, the second-stage product, glyceronitrile **7**, was also detected in the reaction mixture at this stage. Comparing ^13^C NMR spectra at different time points revealed that the bisulfite adducts of the first-stage reduction products, aminomethanesulfonate **10** and hydroxymethanesulfonate **11**, were gradually converted to the first-stage homologation products, glycolonitrile **6** and glycine nitrile **14** as the bisulfite and sulfite in the mixture were consumed.

Our initial experiments with HCN **1** and bisulfite/sulfite had simulated the delivery of SO_2_ from the atmosphere into groundwater containing cyanide salts derived from the prior thermal metamorphosis of sodium or potassium ferrocyanide salts in the dry-state.^6^ Alternatively, bisulfite and formaldehyde **5**, produced atmospherically by photoreduction of CO_2_,^7^ could have rained-in to cyanide containing groundwater as hydroxymethanesulfonate **11**. We therefore explored the chemistry starting directly from **11** and observed efficient production of glycolonitrile **6** when a solution of **11** was mixed with potassium cyanide.

Starting with an initial ratio of cyanide to bisulfite/sulfite (all in the form of hydroxymethanesulfonate **11**) of 1 : 1, the ratio of glycolonitrile **6** to hydroxymethanesulfonate **11** in the mixture had reached 4 : 1 after equilibration (ESI), liberating free bisulfite/sulfite to act as a reductant in subsequent photochemistry. To determine the extent of Kiliani–Fischer homologation upon irradiation, we again used ^13^C-labelled reagents with analysis by ^13^C NMR spectroscopy (Fig. 1).

**Fig. 1:**
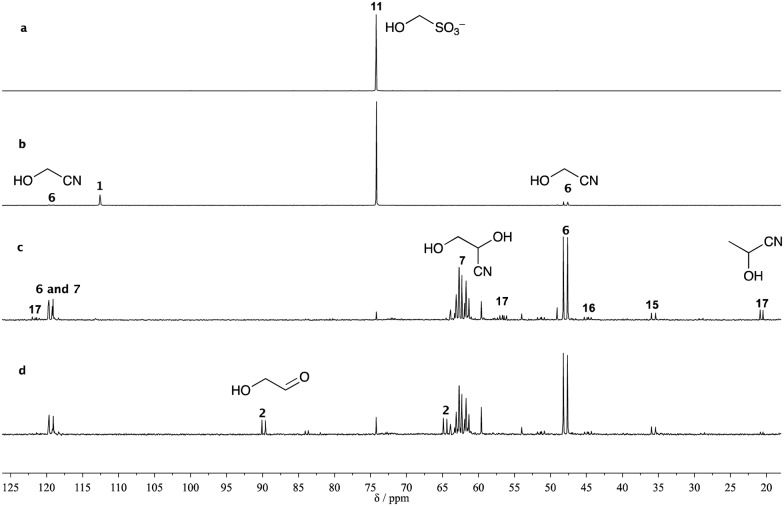
^13^C NMR Spectra of the reaction mixtures with 200 mM ^13^C-labelled KCN, 200 mM Na_2_SO_3_ and 100 mM ^13^C-labelled formaldehyde (in 10% D_2_O in H_2_O). (a) ^13^C-Labelled **11**; (b) as (a), then mixed with ^13^C-labelled KCN and NaH_2_PO_4_ at pH 7; (c) the mixture from (b) after irradiation at 254 nm for 12.5 h; (d) the mixture from (c) after sparging with argon for 13 h.

The equilibration reaction between hydroxymethanesulfonate **11** and cyanide with glycolonitrile **6** and bisulfite/sulfite was mimicked by mixing 1 equivalent of ^13^C-labelled formaldehyde **5** with 2 equivalents of disodium sulfite and 2 equivalents of ^13^C-labelled potassium cyanide and adjusting the pH of the solution to 7. Initially, only hydroxymethanesulfonate **11**, glycolonitrile **6** and excess HCN **1** were observed in the ^13^C NMR spectrum. However, after irradiation for 12 h, nearly all the hydroxymethanesulfonate **11** had been converted into glycolonitrile **6**, glyceronitrile **7**, serine nitrile **16** (convertible to serine by hydrolysis) and iminodiacetonitrile **15**. Interestingly, a small amount of acetaldehyde cyanohydrin **17** (convertible to lactate by hydrolysis) was also produced in the reaction, which could lead to alanine nitrile (convertible to alanine by hydrolysis) if sufficient ammonia was present in the system at a later stage.^2^

We propose that acetaldehyde **18** originates from deoxygenation of glycoaldehyde **2** (Scheme 1) as we had found using H_2_S as the stoichiometric reductant in our earlier work.^2^ To quantify the yields of reduced products, a known amount of ^13^C-labelled sodium formate was added to the solution as an external standard, and the mixture was analyzed by quantitative ^13^C NMR spectroscopy (ESI). Reduced products constituted 34% of the mixture including glyceronitrile **7** (26%), serine nitrile **16** (4%) and acetaldehyde cyanohydrin **17** (4%). Theoretically, reduced products could be obtained in up to 50% yield from a 1 : 1 mixture of cyanide and bisulfite/sulfite, as the reduction of one nitrile group requires two electrons released from two equivalents of sulfite. After sparging argon through the reaction mixture for 13 h to expel HCN **1** from the solution, free glycolaldehyde **2** could be observed in the ^13^C NMR spectrum (Fig. 1d).

In our previous synthesis using H_2_S as the reductant, copper(i) cyanide was found to accelerate the photoreduction of glycolonitrile **6**, providing **2** in 42% yield after 4 h of irradiation.^2^ In comparison, the new photoreduction with sulfite alone as the reductant, gave reduced products in a lower yield with longer irradiation times (12 h), and this raised concerns about its prebiotic plausibility. We therefore looked for an Earth-abundant compound to accelerate the sulfite reduction chemistry.

It is known that photoionization of ferrocyanide ([Fe^II^(CN)_6_]^4–^, ESI 1.2†), effected by UV irradiation at short wavelengths, provides ferricyanide ([Fe^III^(CN)_6_]^3–^) and hydrated electrons.^8^ Indeed we had previously attempted using ferrocyanide for reductive homologation chemistry, but it proved inefficient on its own, which we put down to efficient geminate recombination of the electrons and ferricyanide regenerating ferrocyanide. However, in the context of using sulfite as the stoichiometric reductant, ferrocyanide piqued our interest again because it is known that sulfite reduces ferricyanide to ferrocyanide and, in the process, is converted to sulfate.^9^–^11^ Thus, depending on the relative rates of several processes, added ferrocyanide might double the reducing capacity of sulfite and accelerate the photochemically-driven reductive homologation of HCN **1**. To investigate whether ferrocyanide might act in this way, a solution of 1 equivalent of ^13^C-labelled KCN and 1 equivalent of Na_2_SO_3_ in phosphate buffer was divided in two and 10 mol% K_4_[Fe(CN)_6_] was then added to one portion. The two solutions were then irradiated side-by-side for 3 h (Fig. 2). The reaction mixture lacking ferrocyanide gave only the first-stage reduction products **6**, **10**, **11** and a trace of **13**, while the reaction mixture including ferrocyanide furnished mainly the second-stage reduction product glyceronitrile **7** together with a third-stage reduction product **19**, the cyanohydrin of glyceraldehyde **3**. A similar comparison (ESI) was also made of the reactions starting from mixtures of ^13^C-labelled hydroxymethanesulfonate **11** and ^13^C-labelled HCN **1** with and without added ferrocyanide. In the reaction mixture including ferrocyanide, most of the HCN **1** and the hydroxymethanesulfonate **11** had been consumed within 3 h, providing the reduced product glyceronitrile **7** as well as free glycolaldehyde **2** and the cyanohydrin of glyceraldehyde **19**. By comparison, the reaction mixture lacking ferrocyanide showed considerably less efficient reduction in the same period of time (ESI). In order to quantify the effect of ferrocyanide on the photoreduction, the reaction of hydroxymethanesulfonate **11** with KCN was repeated with unlabelled **11**. Hydroxymethanesulfonate **11** was mixed with 1 equivalent of KCN in phosphate buffer and the resulting mixture was again divided into two parts, into one of which was added 10 mol% K_4_[Fe(CN)_6_]. Reactions were monitored periodically by ^1^H NMR spectroscopy and yields of products were calculated by relative integration of their proton resonance signals (Fig. 3).

**Fig. 2:**
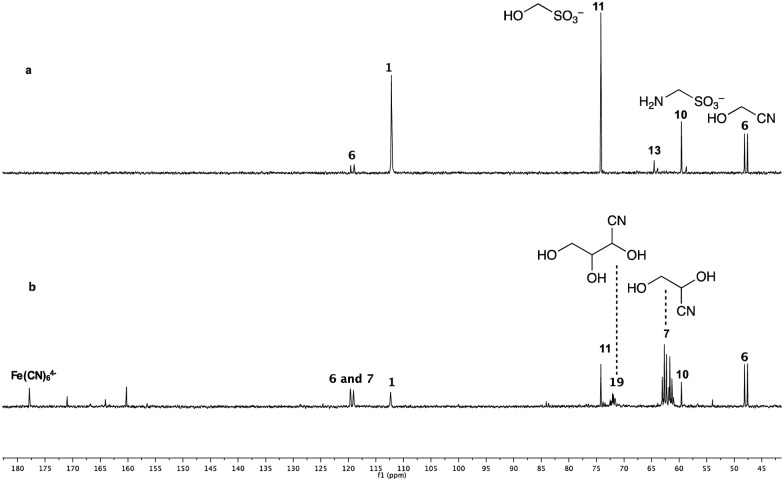
^13^C NMR Spectra of the reaction mixtures with 25 mM ^13^C-labelled KCN, 25 mM Na_2_SO_3_ and 100 mM NaH_2_PO_4_ in D_2_O/H_2_O (10% D_2_O in H_2_O) after irradiation for 3 h. (a) The reaction with no K_4_Fe(CN)_6_; (b) the reaction with 10 mol% K_4_Fe(CN)_6_.

**Fig. 3:**
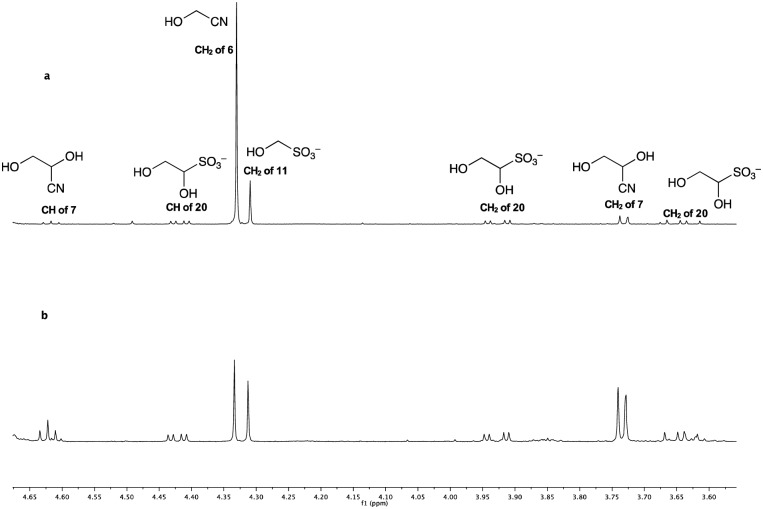
^1^H NMR Spectra of the reaction mixtures with 25 mM of **11**, 25 mM KCN and 100 mM NaH_2_PO_4_ in D_2_O/H_2_O (10% D_2_O in H_2_O) after irradiation for 1 h. (a) The reaction with no K_4_Fe(CN)_6_; (b) the reaction with 10 mol% K_4_Fe(CN)_6_.

Comparing the reactions after only 1 h of irradiation, the catalyzed, or promoted reaction was found to have proceeded rapidly, affording 68% yield of total reduced products (glyceronitrile **7** in 40% yield and glycolaldehyde sulfite adduct **20** in 28% yield), while the control reaction gave only 20% yield of reduced products, eventually increasing to 25% after 3 h.

In the control reaction starting from HCN **1** and sulfite, third-stage reduction products such as glyceraldehyde **3** and its cyanohydrin **19** could barely be detected in the photoreduction mixtures. To investigate the effect of ferrocyanide on the later stages of the overall synthetic scheme, we simply mixed glycolaldehyde **2** with 1 equivalent of KCN and 1 equivalent of Na_2_SO_3_ in phosphate buffer. In the dark, the ratio of the sulfite adduct **20** to cyanohydrin **7** was 2.4 : 1. As before, the mixture was divided into two parts, into one of which was added 10 mol% K_4_[Fe(CN)_6_]. After 1 h of irradiation, the photoreduction reaction including ferrocyanide afforded 30% of **19**, the cyanohydrin of glyceraldehyde and the ratio of **20** to **7** had changed to 0.6 : 1 (ESI). The sulfite in the mixture was efficiently consumed (as reductant) and the equilibrium was in favor of the formation of cyanohydrins **7** and **19**. In comparison, the control reaction afforded no detectable **19** after 1 h of irradiation, but afforded 3% of deoxygenated products (acetaldehyde sulfite adduct **21** and acetaldehyde cyanohydrin **17**) deriving from glycoaldehyde **2**. After 3 h of irradiation, 10% of **19** and 8% of acetaldehyde derivatives were observed. Based on our experimental findings and results from the literature, reaction mechanisms involving cyanoferrate photoredox cycling are proposed here (Scheme 2 and ESI 1.3†).

**Scheme 2:**
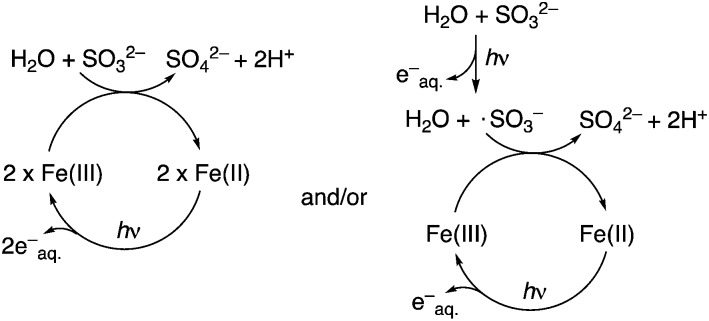
Proposed mechanisms for the photoredox cycling of iron(iii) ⇔ iron(ii) in the presence of Na_2_SO_3_.

In conclusion, through sulfite (SO_3_^2–^) and catalyzed, or promoted by ferrocyanide ([Fe^II^(CN)_6_]^4–^), SO_2_ can act as a more efficient and globally available reductant than H_2_S in the photochemically-driven homologation of HCN **1** to (precursors of) biomolecules. Considering the ready availability of ferrous iron (Fe^II^) on early Earth, the ease with which atmospheric SO_2_ may be concentrated into groundwater, and the numerous mechanisms for supply of HCN, the sulfite-mediated, ferrocyanide-accelerated photoreduction of cyanide offers a synthesis of sugars and precursors of hydroxy acids and amino acids compatible with a globally plausible geochemical scenario.

This work was supported by the Medical Research Council (no. MC_UP_A024_1009 to J. D. S.) and the Simons Foundation (no. 290362 to J. D. S. and no. 290360 to D. D. S.). S. R., Z. R. T. & D. D. S. acknowledge the Harvard Origins of Life Initiative. The authors thank Dr T. Rutherford for assistance with NMR spectroscopy.

## Conflicts of interest

There are no conflicts to declare.

## Supplementary Material

Supplementary information
