# Supplementary material for: Photochemical reductive homologation of hydrogen cyanide using sulfite and ferrocyanide
Source: Chem Commun (Camb). 2018 May 15;54(44):5566–9. doi: 10.1039/c8cc01499j (PMC5972737; doi:10.1039/c8cc01499j)
Supplement: Supplementary file 1 [file CC-054-C8CC01499J-s001.pdf]

Electronic Supplementary Material (ESI) for ChemComm.
This journal is © The Royal Society of Chemistry 2018

Supporting Information

Photochemical reductive homologation of hydrogen cyanide using sulfite and ferrocyanide

Jianfeng Xu,[a] Dougal J. Ritson,[a] Sukrit Ranjan,[b] Zoe R. Todd,[b] Dimitar D. Sasselov[b] and John D. Sutherland*[a]

[a] *MRC Laboratory of Molecular Biology, Francis Crick Avenue, Cambridge Biomedical Campus, Cambridge, CB2 0QH, UK. E-mail: johns@mrc-lmb.cam.ac.uk*
[b] *Harvard-Smithsonian Center for Astrophysics, 60 Garden Street, Cambridge, MA 02138, USA.*

Abstract: Photoredox cycling during UV irradiation of ferrocyanide ($[Fe^{II}(CN)_6]^{4-}$) in the presence of stoichiometric sulfite (SO_3^{2-}) is shown to be an extremely effective way to drive the reductive homologation of hydrogen cyanide (HCN) to simple sugar and amino acid precursors.

Table of Contents

1 Extended discussion 4

1.1 Bisulfite & UV irradiation 4

1.2 Ferrocyanide 4

1.3 Mechanism with sulfite as stoichiometric reductant and ferrocyanide as catalyst/promoter 4

2 General methods 5

Experimental procedures to synthesize standards 5

2.1 Aminomethanesulfonate (10), hydroxymethanesulfonate (11) and iminodimethanesulfonate (13). 5

2.2 Aminomethanedisulfonate (12) [1] 6

2.3 1,2-Dihydroxypropanenitrile (7) 6

2.4 1-^{13}C-2-amino-3-hydroxypropanenitrile (16) 6

2.5 2-Hydroxypropanenitrile (17) and 1-hydroxyethane-1-sulfonate (21) 6

2.6 2,3,4-trihydroxybutanenitrile (19) 7

2.7 1,2-Dihydroxyethane-1-sulfonate (20) 7

3 Experimental procedures for UV experiments 7

3.1 Photoreduction of ^{13}C-labelled KCN with sodium sulfite. 7

3.2 Photoreduction of ^{13}C-labelled hydroxymethanesulfonate (11) with ^{13}C-labelled KCN ... 7

3.3 Photoreduction of ^{13}C-labelled hydroxymethanesulfonate (11) with ^{13}C-labelled KCN with or without ferrocyanide 8

3.4 Photoreduction of hydroxymethanesulfonate (11) with KCN with or without ferrocyanide. 8

3.5 Photoreduction of 1,2-Dihydroxypropanenitrile (7) with Na_2SO_3 with or without ferrocyanide 8

4. NMR spectra 9

4.1 NMR spectra for synthetic standards 9

4.1.1 ^{1}H NMR spectrum of ^{13}C-labelled hydroxymethanesulfonate 11 in D_2O/H_2O 9

4.1.2 ^{13}C NMR spectrum of ^{13}C-labelled hydroxymethanesulfonate 11 in D_2O/H_2O 10

4.1.3 ^{1}H NMR spectrum of ^{13}C-labelled aminomethanesulfonate (10) and iminodimethanesulfonate (13) in D_2O/H_2O 10

4.1.4 ^{13}C NMR spectrum of ^{13}C-labelled aminomethanesulfonate (10) and iminodimethanesulfonate (13) in D_2O/H_2O 11

4.1.5 ^{13}C NMR spectrum of ^{13}C-labelled aminomethanedisulfonate (12) (mixed with excess HCN/CN^-) in D_2O/H_2O 11

4.1.6 ^{1}H NMR spectrum of 1,2-dihydroxypropanenitrile (7) (mixed with residual glycoaldehyde 2) in D_2O/H_2O 12

4.1.7 ^{13}C NMR spectrum of 1,2-dihydroxypropanenitrile (7) (mixed with residual glycoaldehyde 2) in D_2O/H_2O 12

4.1.8 ^{13}C NMR spectrum of 1-^{13}C-2-amino-3-hydroxypropanenitrile (16) (mixed with ^{13}C-1,2-dihydroxypropane nitrile (7)) in D_2O/H_2O 13

4.1.9 ^{1}H NMR spectrum of 2-hydroxypropanenitrile (17) in D_2O/H_2O 13

4.1.10 ^{13}C NMR spectrum of 2-hydroxypropanenitrile (17)(mixed with remaining HCN/CN^-) in D_2O/H_2O 14

4.1.11 ^{1}H NMR spectrum of 2,3,4-trihydroxybutanenitrile (19) in D_2O/H_2O 14

4.1.12 ^{13}C NMR spectrum of 2,3,4-trihydroxybutanenitrile (19) in D_2O/H_2O 15

4.1.13 ^{1}H NMR spectrum of 1,2-dihydroxyethane-1-sulfonate (20) in D_2O/H_2O 16

4.1.14 ^{13}C NMR spectrum of 1,2-dihydroxyethane-1-sulfonate (20) in D_2O/H_2O 16

4.1.15 ^{1}H NMR spectrum of 1-hydroxyethane-1-sulfonate (21) in D_2O/H_2O 17

4.1.16 ^{13}C NMR spectrum of 1-hydroxyethane-1-sulfonate (21) in D_2O/H_2O 17

4.2 NMR spectra for UV photoreduction 18

4.2.1 ^{13}C NMR time course of the photoreduction of ^{13}C-labelled KCN with sodium sulfite 18

4.2.2 ^{13}C NMR spectra of the spiking experiments for the photoreduction of ^{13}C-labelled KCN with sodium sulfite 19

4.2.3 ^{13}C NMR spectra of the spiking experiments for the photoreduction of ^{13}C-labelled hydroxymethanesulfonate (11) with ^{13}C-labelled KCN 20

4.2.4 Quantitative ^{13}C NMR spectrum of the photoreduction of ^{13}C-labelled hydroxymethanesulfonate (11) with ^{13}C-labelled KCN 21

4.2.5 ^{13}C NMR spectra of the photoreductions of ^{13}C-labelled hydroxymethanesulfonate (11) with ^{13}C-labelled KCN with or without ferrocyanide 22

4.2.6 ^{1}H NMR time course of the photoreductions of hydroxymethanesulfonate (11) with KCN with or without ferrocyanide 23

4.2.7 ^{1}H NMR time course of the photoreduction of 1,2-dihydroxypropanenitrile (7) with sodium sulfite with or without ferrocyanide 24

References 24

1 Extended discussion

1.1 Bisulfite & UV irradiation

Ultraviolet light likely played an important role in chemistry on the surface of early Earth,[1-5] and we (J. X., D. J. R. & J. D. S.) and others have frequently used UV light from a mercury lamp with primary emission at 254 nm to investigate potentially prebiotic photochemistry. Although early solar UV emission was broadband, its influx on the earth would have been constrained due to absorption by atmospheric gases. UV wavelengths below 204 nm would have been persistently screened out due to absorption by atmospheric CO_2 and H_2O vapour, and SO_2 and H_2S released by volcanism would have transiently acted as additional UV shields during and shortly after periods of volcanism.[6] In high concentrations, H_2S strongly absorbs UV wavelengths around 200 nm while SO_2 absorbs around 200 nm and 290 nm. Interestingly, neither species strongly absorbs around 250 nm, meaning that our UV source emits at a wavelength that would have been accessible across a broad range of H_2S and SO_2 abundances. Although this UV photochemistry is not recapitulated in extant biology, it could have served to lay down supplies of (proto)biomolecules to kick-start life and provision it in its early stages. Biology could then learn different routes to resupply these molecules when prebiotic stores ran out. Biology uses chemistry catalysed by enzymes which is easy to control (by regulating the amount or activity of the enzymes) and tends not to use reaction mechanisms that proceed efficiently in the absence of catalysis. Thus, one would expect biology to make the same products as prebiotic chemistry, but using different transformations or the same transformations using different mechanisms to prebiotic chemistry.

Phosphate was used as a buffer in this study as its presence in numerous (proto)biomolecules (eg. RNA, lipids, ATP and nucleotide cofactors) implies prebiotic and early biotic availability.

The high levels of sulfate in Martian soils strongly suggests that volcanic emissions of SO_2 and H_2S were common on early Mars.[7,8] Volcanism is still the major source of atmospheric SO_2 and H_2S on Earth today and it is plausible that volcanic activity was, at least intermittently, much higher on anoxic early Earth, allowing SO_2 to reach higher concentrations.[9] These considerations suggest that SO_2 should be considered as an alternative to H_2S as an atmospherically-sourced reductant for prebiotic chemistry. Compared to H_2S, SO_2 is easily concentrated from the atmosphere into groundwater due to the favourability of its hydration and the acidity of the hydrate (pK_a = 1.8 for 'sulfurous acid' ($SO_2 \cdot xH_2O$), pK_a = 7.2 for bisulfite (HSO_3^-)).[7,8] Lastly, it is known from EPR studies [10-12] that UV irradiation of aqueous solutions of sulfite (SO_3^{2-}) produces sulfite radical anions ($^{\bullet}SO_3^-$) and hydrated electrons.

1.2 Ferrocyanide

Ferrocyanide ($[Fe^{II}(CN)_6]^{4-}$) has been considered as a prebiotically plausible solution phase repository of HCN **1** delivered from the atmosphere of early Earth.[13] Free cyanide can be released from solutions of the complex by photoaquation at longer UV wavelengths,[14,15] or, more efficiently, by thermal decomposition of sodium or potassium ferrocyanides in the solid state.[16]

1.3 Mechanism with sulfite as stoichiometric reductant and ferrocyanide as catalyst/promoter

Ferrocyanide ($[Fe(CN)_6]^{4-}$) is photoionized (oxidized) to give ferricyanide ($[Fe(CN)_6]^{3-}$) and a solvated electron as

reported in the literature.[17] Assisted by general acids (phosphate monoanion or bisulfite) the hydrated electrons add to HCN **1** or, later on, to the nitrile groups of cyanohydrins to give reduced products. The ferricyanide is then reduced to ferrocyanide by sulfite, affording sulfate (SO_4^{2-}) as a co-product. Additionally, sulfite might act as a direct source of hydrated electrons during irradiation in which case the sulfite radical anions ($^{\bullet}SO_3^-$) could be oxidised by ferricyanide to sulfate. Whatever the relative contributions of the pathways operative, the sulfite effectively functions as a two-electron donor in the presence of ferrocyanide.[18-20] However, absent ferrocyanide, the reaction proceeds less efficiently and sulfite can only act as a one-electron reductant – the resultant sulfite radical anion ($^{\bullet}SO_3^-$) now dimerising to $S_2O_6^{2-}$ in the process (see inset box in Scheme 1 in the main text).[10-12] The proposed mechanism is also supported by the quantitative data obtained by comparison of the reactions of **11** with 1 equivalent of KCN with and without ferrocyanide (Fig. 3). The reaction involving ferrocyanide afforded a 68% yield (i.e. more than 50%) of reduced products after 1 h of irradiation, which is only stoichiometrically possible with sulfite as a two-electron reductant. The reaction without ferrocyanide could theoretically afford up to 50% of reduced products, however we found that it gave a maximum yield of only 25% of reduced products after 3 h of irradiation.

2 General methods

All reagents and deuterated solvents used for reactions and spiking experiments were purchased from Sigma-Aldrich or Acros Organics and were used without further purification. All photochemical reactions were carried out in *Norell* Suprasil quartz NMR tubes or Spectrosil quartz cuvettes purchased from Sigma-Aldrich using Hg lamps with principal emission at 254 nm in a *Rayonet* photochemical chamber reactor RPR-200, acquired from The Southern New England Ultraviolet Company. A *Mettler Toledo* SevenEasy pH Meter S20 was used to monitor the pH, and deoxygenation of solution was achieved by sparging anhydrous argon through the solution for 15-20 min. All unknown compounds in the reaction mixtures were confirmed by spiking experiments with authentic compounds either purchased from Sigma-Aldrich or synthesized in house using conventional synthetic chemistry. ^{1}H and ^{13}C NMR spectra were acquired using a *Bruker* Ultrashield 400 Plus operating at 400.1 MHz and 100.6 MHz respectively. Samples consisting of H_2O/D_2O mixtures were analyzed using HOD suppression to collect ^{1}H NMR data. The quantitative ^{13}C NMR spectra were acquired with inverse-gated decoupling, with a 90˚ excitation pulse and an inter-pulse delay of 70 seconds. ^{13}C longitudinal relaxation time constants (T1) were measured to be no greater than 13 seconds for any of the ^{13}C resonances at 100.6 MHz. Chemical shifts (δ) are shown in ppm. The conversion yields were determined by relative integrations of the signals using a known amount of ^{13}C-labelled sodium formate as internal reference in the ^{1}H NMR spectrum and the quantitative ^{13}C NMR spectrum. Coupling constants (*J*) are given in Hertz and the notations s, d, m represent the multiplicities singlet, doublet, and multiplet signal.

Experimental procedures to synthesize standards

2.1 Aminomethanesulfonate (10), hydroxymethanesulfonate (11) and iminodimethanesulfonate (13).

8.0 μl of ^{13}C-labelled formaldehyde solution (0.05 mmol, 20 wt. % in water, from Sigma-Aldrich) was mixed with 6 mg of sodium sulphite (0.05 mmol) in 0.5 mL of degassed water (containing 10% of D_2O). The pH of the mixture

was adjusted to 7 with 1M HCl before being transferred to an NMR tube to record the ^1H and ^{13}C NMR spectra of hydroxymethanesulfonate **11**. 50.0 µl of 2M NH_4Cl buffer was added to the mixture, then the pH was adjusted to 9.2. The mixture was kept at room temperature overnight to obtain the ^1H and ^{13}C NMR spectra of aminomethanesulfonate **10** and iminodimethanesulfonate **13** as a mixture. The structures of **10** and **11** were also confirmed by comparison with authentic standards purchased from Sigma – Aldrich. **10**: ^1H NMR (400 MHz, D_2O/H_2O = 1:9): δ 3.64 (d, J ^{13}C-H = 146 Hz); ^{13}C NMR (101 MHz, D_2O/H_2O = 1:9): 59.5. **11**: ^1H NMR (400 MHz, D_2O/H_2O = 1:9): δ 4.34 (d, J ^{13}C-H = 153 Hz); ^{13}C NMR (101 MHz, D_2O/H_2O = 1:9): δ 74.2. **13**: ^1H NMR (400 MHz, D_2O/H_2O = 1:9): δ 3.95 (d, J ^{13}C-H = 146 Hz); ^{13}C NMR (101 MHz, D_2O/H_2O = 1:9): δ 64.5.

2.2 Aminomethanedisulfonate (12) [21]

7.0 mg of ^{13}C-labelled KCN (0.10 mmol) was mixed with 13.0 mg of Na_2SO_3 (0.10 mmol) in 0.5 mL of degassed water (containing 10% of D_2O). The pH of the mixture was adjusted to 7 with 1M HCl. The mixture was heated at 50 °C for 10 h, then a ^{13}C NMR spectrum was recorded. ^{13}C NMR (101 MHz, D_2O/H_2O = 1:9): δ 81.1.

2.3 1,2-Dihydroxypropanenitrile (7)

15.0 mg of glycoaldehyde dimer $\mathbf{2}_2$ (0.13 mmol) was mixed with 16.0 mg of potassium cyanide (0.25 mmol) in 0.5 mL of degassed water (containing 10% of D_2O). The pH of the mixture was adjusted to 7 with 1M HCl before being transferred to an NMR tube to record the ^1H and ^{13}C NMR spectra of 1,2-dihydroxypropanenitrile **7**. ^1H NMR (400 MHz, D_2O/H_2O = 1:9): δ 4.58 (1H, t, J = 4.8 Hz), 3.68 (2H, dd, J_1 = 4.7 Hz, J_2 = 0.7 Hz; ^{13}C NMR (101 MHz, D_2O/H_2O = 1:9): δ 119.6, 62.8, 61.8.

2.4 1-^{13}C-2-amino-3-hydroxypropanenitrile (16)

12.0 mg of glycoaldehyde dimer $\mathbf{2}_2$ (0.10 mmol) was mixed with 13.2 mg of ^{13}C-labelled potassium cyanide (0.20 mmol) in 0.5 mL of degassed water (containing 10% of D_2O). The pH of the mixture was adjusted to 7 with 1M HCl, followed by addition of 100 µl of 2 M NH_4Cl/NH_3 buffer at pH 9. The mixture was kept at pH 9 overnight, then transferred to an NMR tube to record the ^{13}C NMR spectra of 1-^{13}C-2-amino-3-hydroxypropanenitrile **16**. ^{13}C NMR (101 MHz, D_2O/H_2O = 1:9): δ 121.3, 62.9, 44.9 (J c-c = 57.6 Hz).

2.5 2-Hydroxypropanenitrile (17) and 1-hydroxyethane-1-sulfonate (21)

14.0 µl of acetaldehyde **18** (0.25 mmol) was mixed with 31.5 mg of Na_2SO_3 (0.25 mmol) in 0.5 mL of degassed water (containing 10% of D_2O). The pH of the mixture was adjusted to 7 with 1M HCl before being transferred to an NMR tube to record the ^1H and ^{13}C NMR spectra of 1-hydroxyethane-1-sulfonate **21**. 16.3 mg of KCN (0.25 mmol) was then added to the mixture, and the pH was adjusted to 9.2. The ^1H and ^{13}C NMR spectra of 2-hydroxypropanenitrile **17** were recorded immediately after mixing. **21**: ^1H NMR (400 MHz, D_2O/H_2O = 1:9): δ 4.48 (1H, q, J = 6.5 Hz), 1.37 (3H, d, J = 6.5); ^{13}C NMR (101 MHz, D_2O/H_2O = 1:9): δ 80.4, 17.0. **17**: ^1H NMR (400

MHz, D_2O/H_2O = 1:9): δ 4.67 (1H, q, J = 6.9 Hz), 1.48 (3H, d, J = 6.9); ^{13}C NMR (101 MHz, D_2O/H_2O = 1:9): δ 121.8, 56.6, 20.8.

2.6 2,3,4-trihydroxybutanenitrile (19)

20.0 mg of glyceraldehyde **3** (0.22 mmol) was mixed with 14.3 mg (0.22 mmol) of KCN in 0.5 mL of water (containing 10% of D_2O). The pH of the mixture was adjusted to 7 with 1M HCl, then ^{1}H NMR and ^{13}C NMR spectra were recorded. ^{1}H NMR (400 MHz, D_2O/H_2O = 1:9): δ 4.65 (1H, d, J = 6.0 Hz), 3.90 (1H, m), 3.68 (2H, m); ^{13}C NMR (101 MHz, D_2O/H_2O = 1:9): δ 119.5,119.4, 72.2, 72.0, 62.5, 62, 61.6, 61.3.

2.7 1,2-Dihydroxyethane-1-sulfonate (20)

12.0 mg of glycoaldehyde dimer $\mathbf{2}_2$ (0.1 mmol) was mixed with 25.0 mg of Na_2SO_3 (0.20 mmol) in 0.5 mL of degassed water (containing 10% of D_2O). The pH of the mixture was adjusted to 7 with 1M HCl before it was transferred to an NMR tube to record the ^{1}H and ^{13}C NMR spectra of 1,2-dihydroxyethane-1-sulfonate **20**. ^{1}H NMR (400 MHz, D_2O/H_2O = 1:9): δ 4.48 (1H, dd, J_1 = 8.0 Hz, J_2 = 3.2 Hz), 3.98 (1H, dd, J_1 = 12.1 Hz, J_2 = 3.2 Hz), 3.68 (1H, dd, J_1 = 12.1 Hz, J_2 = 8.0 Hz); ^{13}C NMR (101 MHz, D_2O/H_2O = 1:9): δ 83.9, 61.5.

3 Experimental procedures for UV experiments

3.1 Photoreduction of ^{13}C-labelled KCN with sodium sulfite.

8.0 mg of ^{13}C-labelled KCN (0.12 mmol) was mixed with 15.0 mg of Na_2SO_3 (0.12 mmol) and 15.0 mg of NaH_2PO_4 (0.12 mmol) in 0.5 mL of degassed water (containing 10% of D_2O). The pH of the mixture was adjusted to 7 with 1M HCl. The mixture was subject to irradiation at 254 nm for 5 h. The pH of the mixture was adjusted to 9.2 with 1M NaOH and the mixture was kept at room temperature overnight before being spiked with authentic standards.

3.2 Photoreduction of ^{13}C-labelled hydroxymethanesulfonate (11) with ^{13}C-labelled KCN

8.0 μl of ^{13}C-labelled formaldehyde solution (0.05 mmol, 20 wt. % in water, from Sigma-Aldrich) was mixed with 12.6 mg of Na_2SO_3 (0.10 mmol) in 0.5 mL of degassed water (containing 10% of D_2O). The pH of the mixture was adjusted to 7 with 1M HCl. 6.6 mg of ^{13}C-labelled KCN (0.10 mmol) and 12.0 mg of NaH_2PO_4 were added to the mixture. The pH was readjusted to 7 with 1M HCl. The mixture was transferred to a quartz NMR tube and irradiated at 254nm for 12.5 h in total. The sample was taken out of the photochemical reactor at intervals to record ^{1}H and ^{13}C NMR spectra. At the end of the reaction, 2 mg (0.03 mmol) of ^{13}C-labelled sodium formate was added to the mixture to enable quantification of the reaction products in quantitative ^{13}C NMR spectra. Argon was sparged through the solution at the end to remove excess HCN from the mixture.

3.3 Photoreduction of ^{13}C-labelled hydroxymethanesulfonate (11) with ^{13}C-labelled KCN with or without ferrocyanide

11.2 µl of a solution of ^{13}C-labelled formaldehyde (0.08 mmol, 20 wt. % in water, from Sigma-Aldrich) was mixed with 9.5 mg of Na_2SO_3 (0.08 mmol) in 3.0 mL of degassed water (containing 10% of D_2O). The pH of the mixture was adjusted to 7 with 1M HCl. 5.0 mg of ^{13}C-labelled KCN (0.08 mmol) and 36.0 mg of NaH_2PO_4 (0.30 mmol) were added to the mixture. The pH was readjusted to 7 with 1M HCl. The mixture was divided into two parts, into one of which was added 1.6 mg of $K_4Fe(CN)_6.3H_2O$ (0.004 mmol, 10 mol%). Both solutions in quartz NMR tubes were irradiated in the photochemical reactor side by side and taken out at intervals to record ^{13}C spectra.

3.4 Photoreduction of hydroxymethanesulfonate (11) with KCN with or without ferrocyanide.

7.0 mg of hydroxymethanesulfonate **11** (0.05 mmol), 3.3 mg of KCN (0.05 mmol) and 24.0 mg of NaH_2PO_4 (0.20 mmol) were mixed in 2.0 mL of degassed water (containing 10% of D_2O). The pH of the mixture was adjusted to 7 with 1M HCl. The solution was divided into two portions, into one of which was added 1.0 mg of $K_4Fe(CN)_6.3H_2O$ (0.003 mmol,10 mol%). Both solutions in quartz NMR tubes were irradiated in the photochemical reactor side by side and taken out at intervals to record ^{1}H spectra.

3.5 Photoreduction of 1,2-Dihydroxypropanenitrile (7) with Na_2SO_3 with or without ferrocyanide

4.5 mg of glycoaldehyde dimer $\mathbf{2}_2$ (0.08 mmol for **2**) was mixed with 5.0 mg of KCN (0.08 mmol) in 3.0 mL of degassed water (containing 10% of D_2O) at pH 7 to obtain 1,2-dihydroxypropanenitrile *in situ*. To the mixture was added 9.5 mg of sodium sulfite (0.08 mmol) and 36.0 mg of NaH_2PO_4 (0.30 mmol). The pH of the solution was readjusted to 7 with 1M HCl or 1 M NaOH. The solution was divided into two portions, into one of which was added 1.6 mg of $K_4Fe(CN)_6.3H_2O$ (0.004 mmol,10 mol%). Both solutions in quartz NMR tubes were irradiated in the photochemical reactor side by side for 3 h in total and taken out at intervals to record ^{1}H spectra.

4. NMR spectra

4.1 NMR spectra for synthetic standards

4.1.1 ^{1}H NMR spectrum of ^{13}C-labelled hydroxymethanesulfonate 11 in D_2O/H_2O

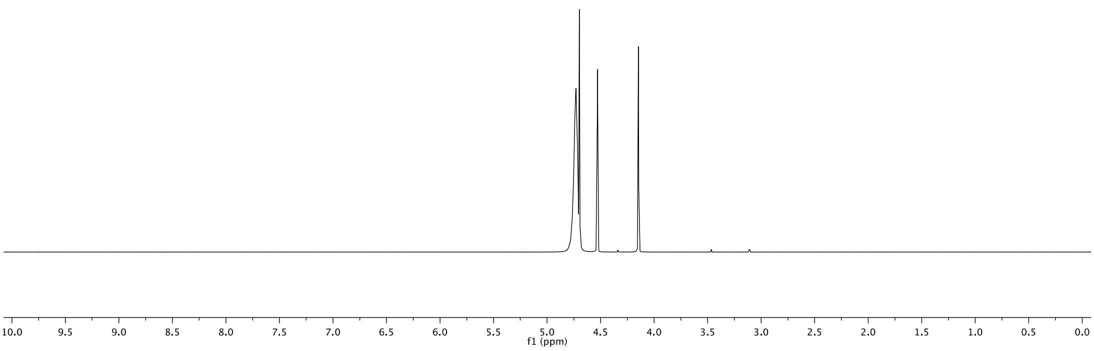

4.1.2 ^{13}C NMR spectrum of ^{13}C-labelled hydroxymethanesulfonate 11 in D_2O/H_2O

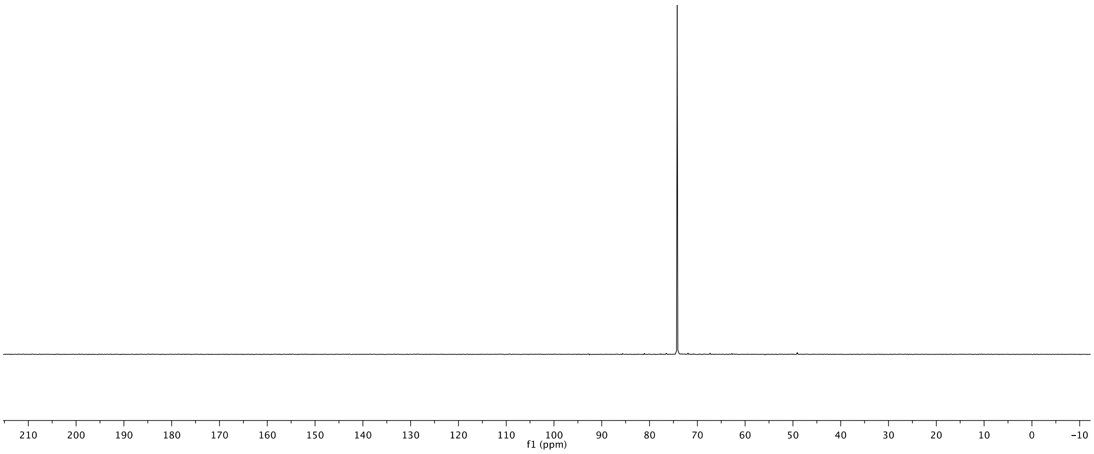

4.1.3 ^{1}H NMR spectrum of ^{13}C-labelled aminomethanesulfonate (10) and iminodimethanesulfonate (13) in D_2O/H_2O

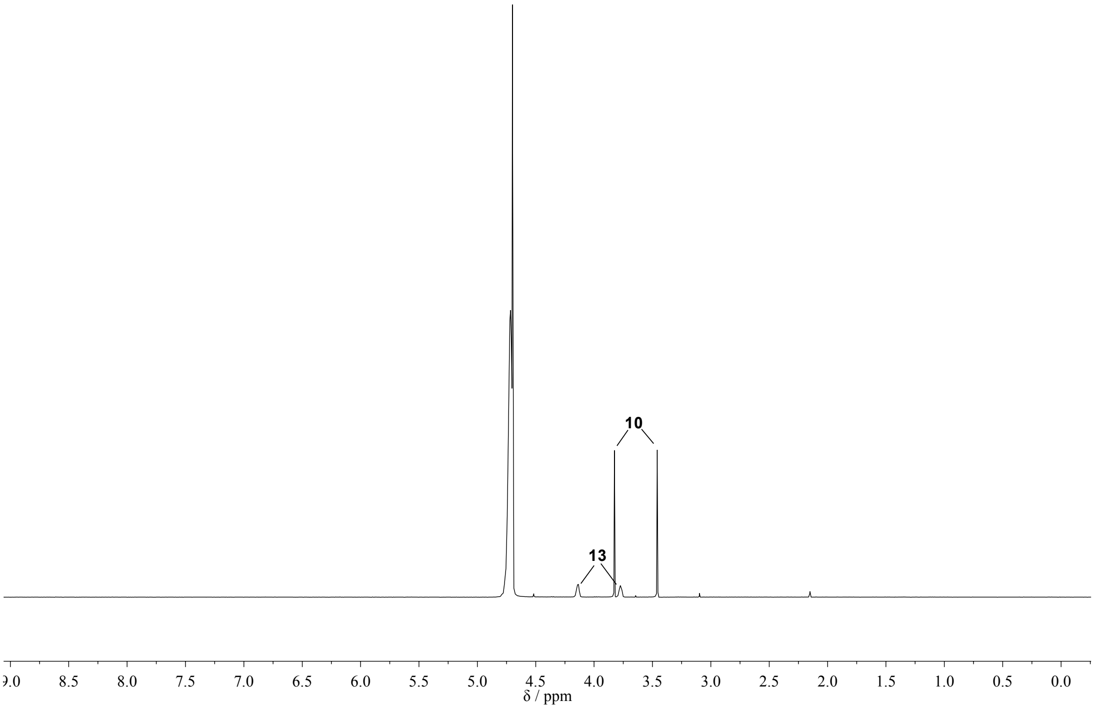

4.1.4 ^{13}C NMR spectrum of ^{13}C-labelled aminomethanesulfonate (10) and iminodimethanesulfonate (13) in D_2O/H_2O

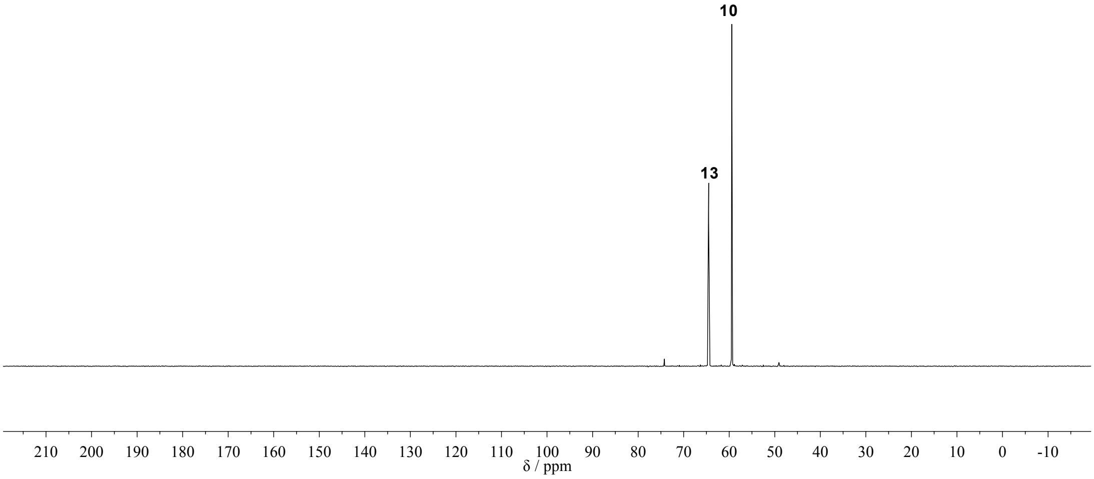

4.1.5 ^{13}C NMR spectrum of ^{13}C-labelled aminomethanedisulfonate (12) (mixed with excess HCN/CN^-) in D_2O/H_2O

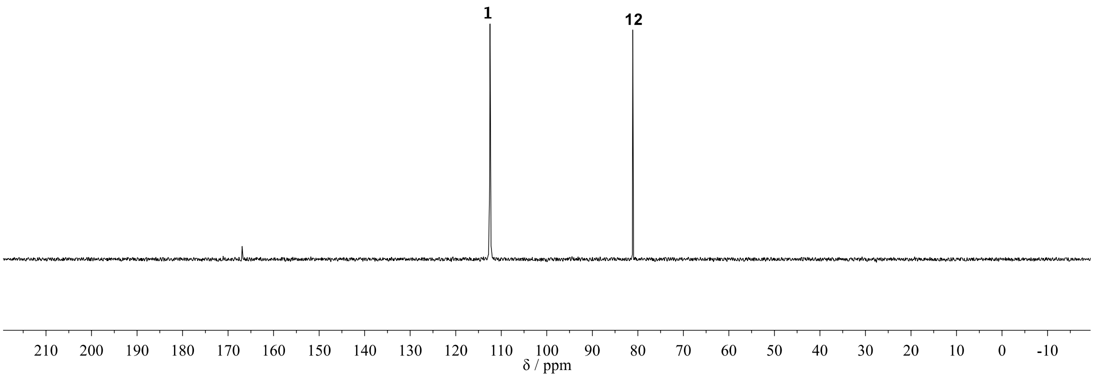

4.1.6 ^{1}H NMR spectrum of 1,2-dihydroxypropanenitrile (7) (mixed with residual glycoaldehyde 2) in D_2O/H_2O

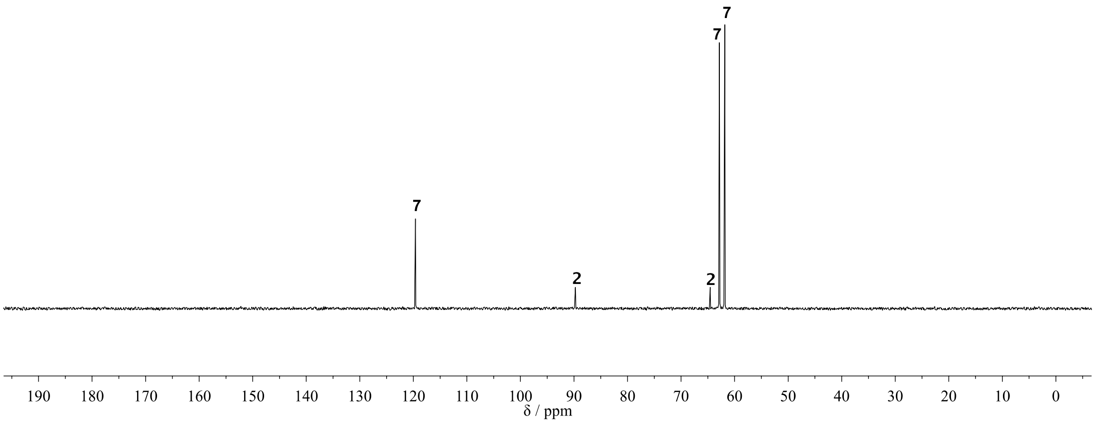

4.1.7 ^{13}C NMR spectrum of 1,2-dihydroxypropanenitrile (7) (mixed with residual glycoaldehyde 2) in D_2O/H_2O

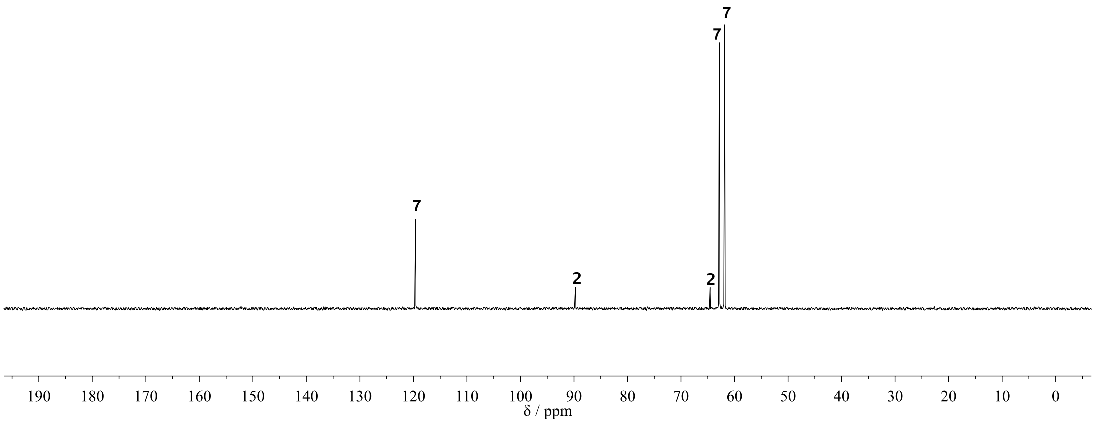

4.1.8 ^{13}C NMR spectrum of 1-^{13}C-2-amino-3-hydroxypropanenitrile (16) (mixed with ^{13}C-1,2-dihydroxypropane nitrile (7)) in D_2O/H_2O

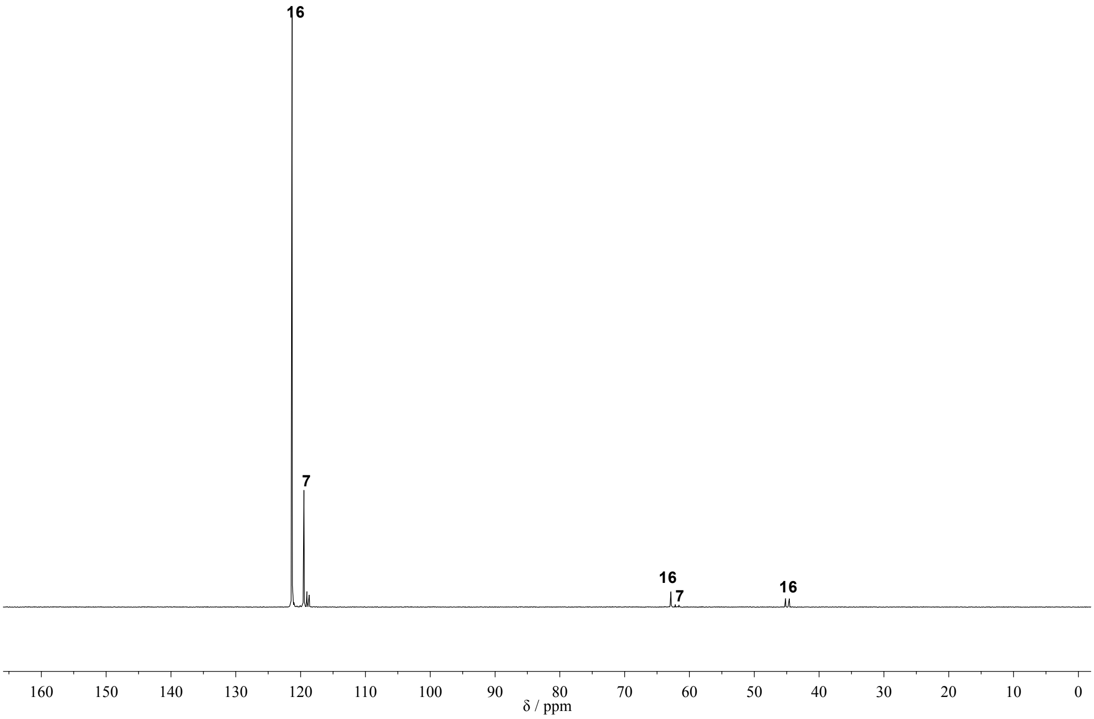

4.1.9 ^{1}H NMR spectrum of 2-hydroxypropanenitrile (17) in D_2O/H_2O

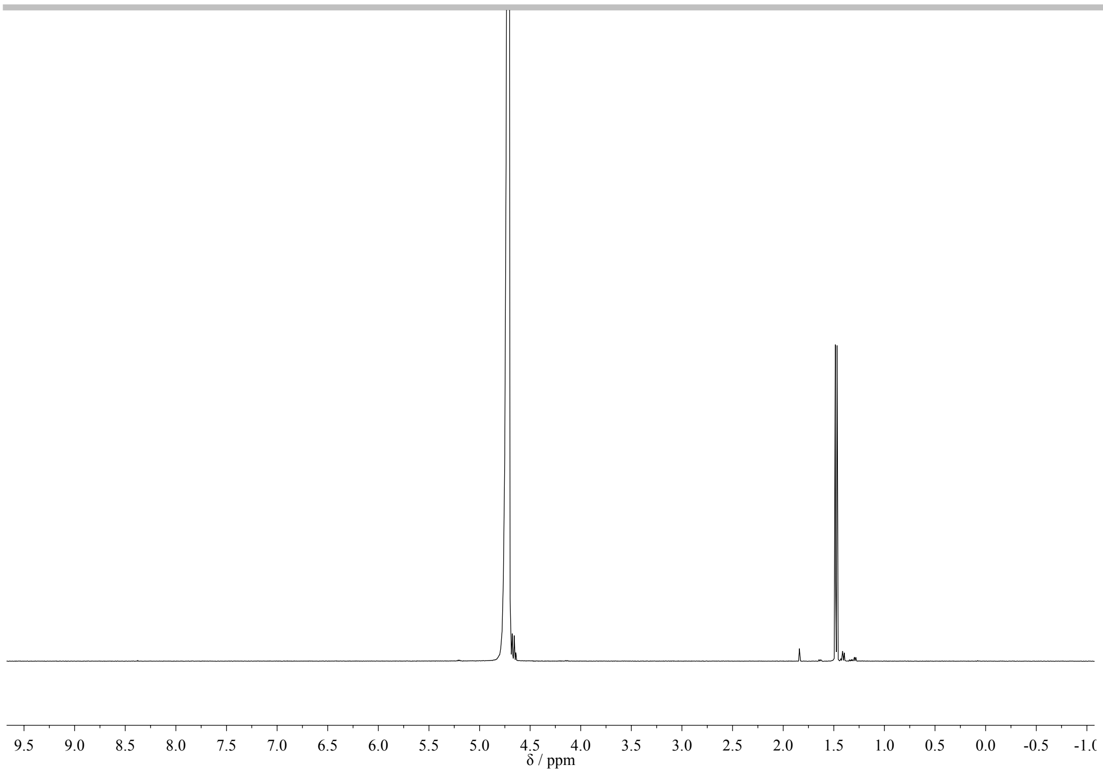

4.1.10 ^{13}C NMR spectrum of 2-hydroxypropanenitrile (17)(mixed with remaining HCN/CN^-) in D_2O/H_2O

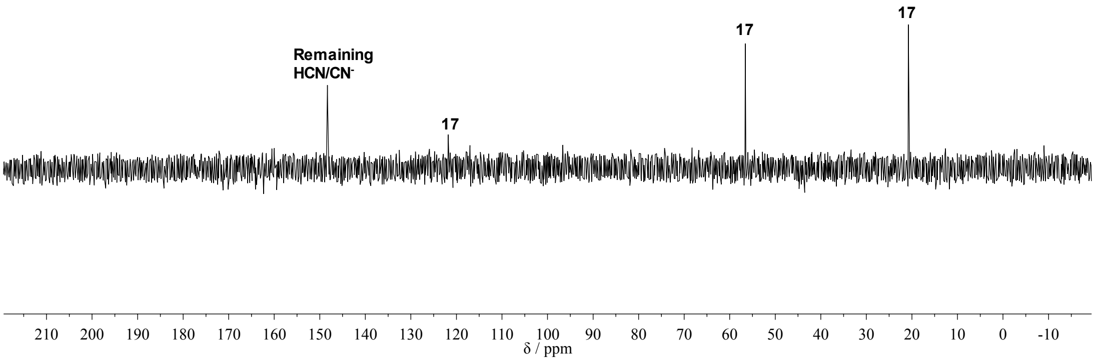

4.1.11 ^1H NMR spectrum of 2,3,4-trihydroxybutanenitrile (19) in D_2O/H_2O

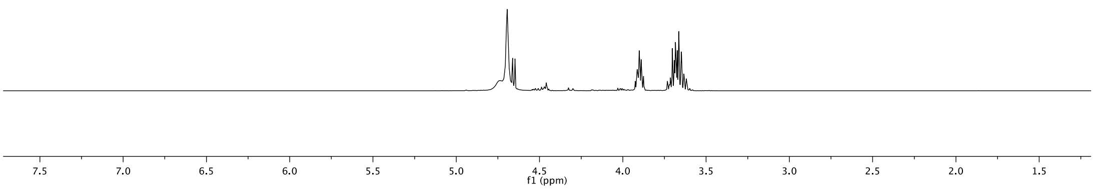

4.1.12 ^{13}C NMR spectrum of 2,3,4-trihydroxybutanenitrile (19) in D_2O/H_2O

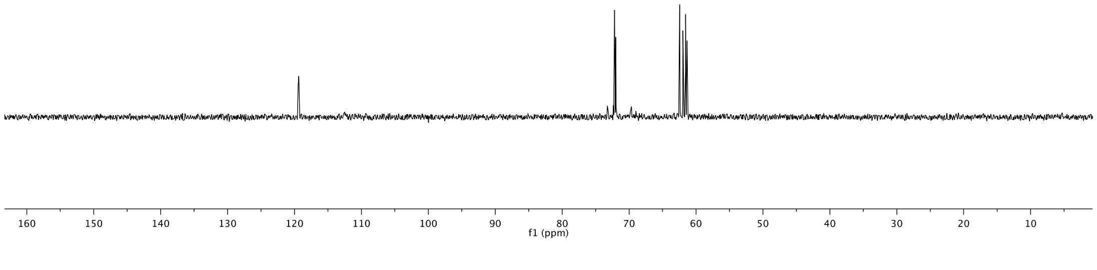

4.1.13 ^{1}H NMR spectrum of 1,2-dihydroxyethane-1-sulfonate (20) in D_2O/H_2O

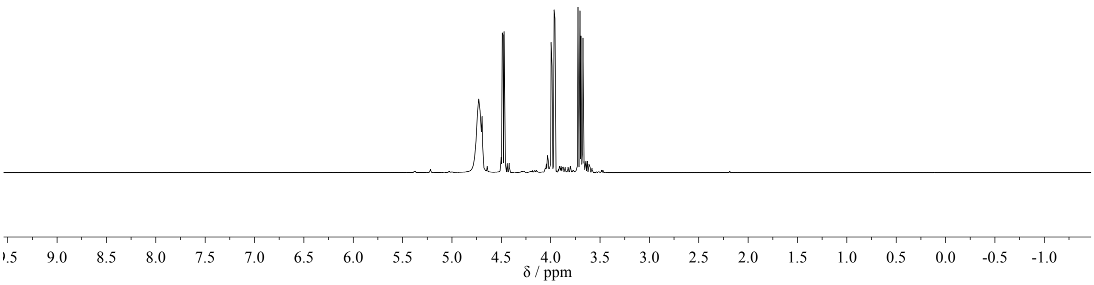

4.1.14 ^{13}C NMR spectrum of 1,2-dihydroxyethane-1-sulfonate (20) in D_2O/H_2O

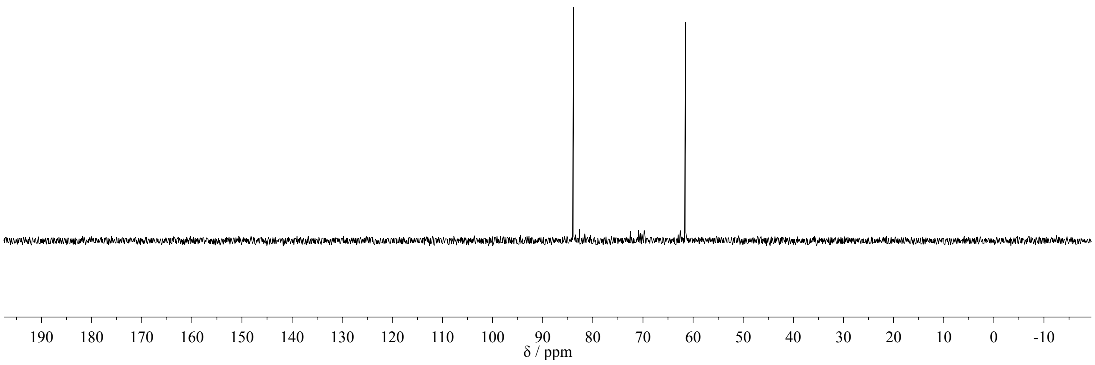

4.1.15 ^{1}H NMR spectrum of 1-hydroxyethane-1-sulfonate (21) in D_2O/H_2O

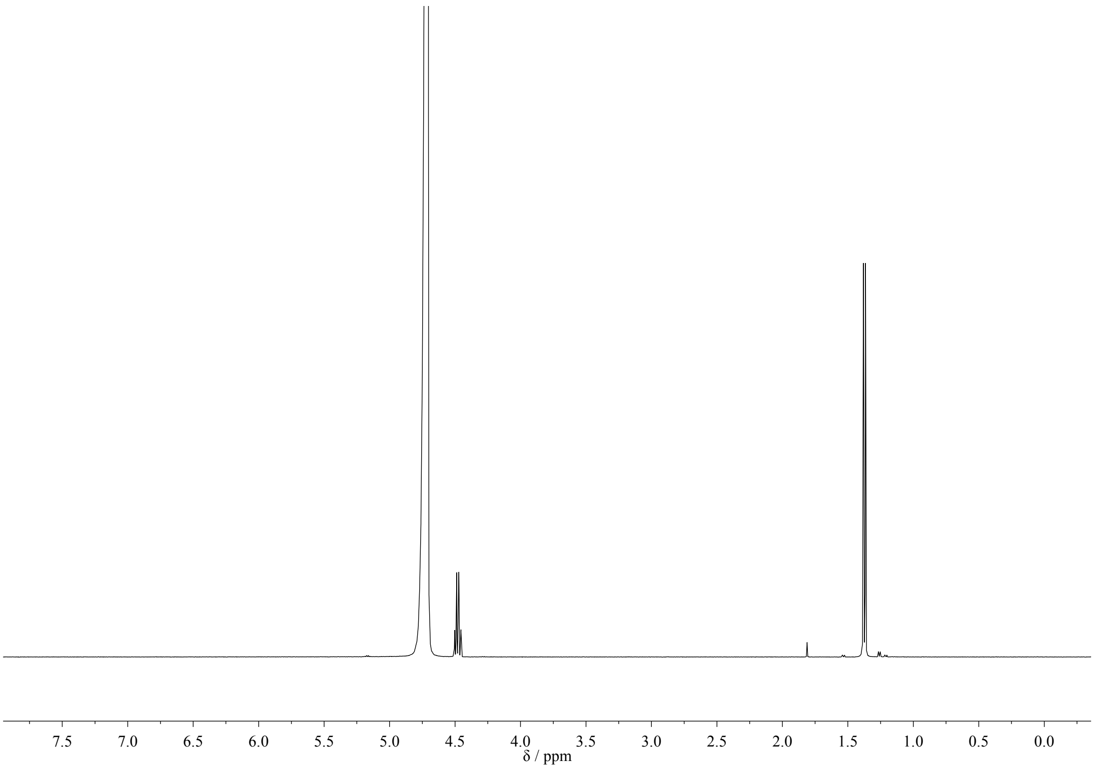

4.1.16 ^{13}C NMR spectrum of 1-hydroxyethane-1-sulfonate (21) in D_2O/H_2O

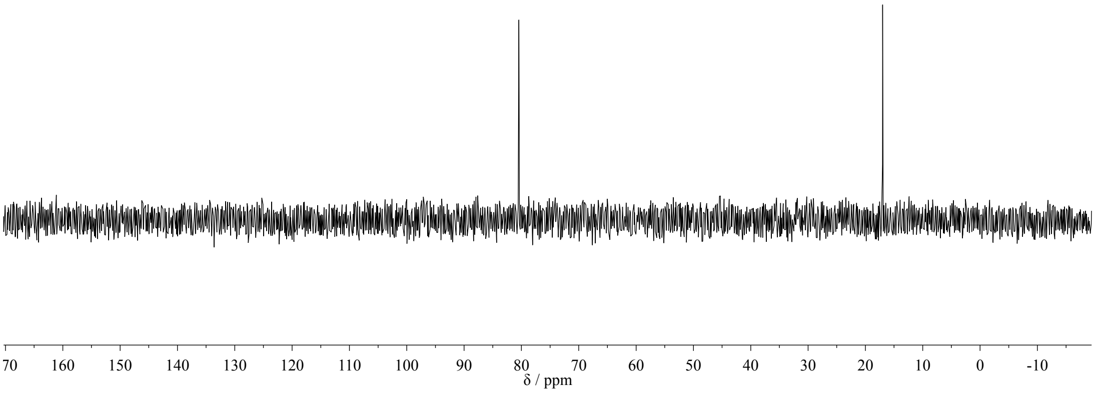

4.2 NMR spectra for UV photoreduction

4.2.1 ^{13}C NMR time course of the photoreduction of ^{13}C-labelled KCN with sodium sulfite

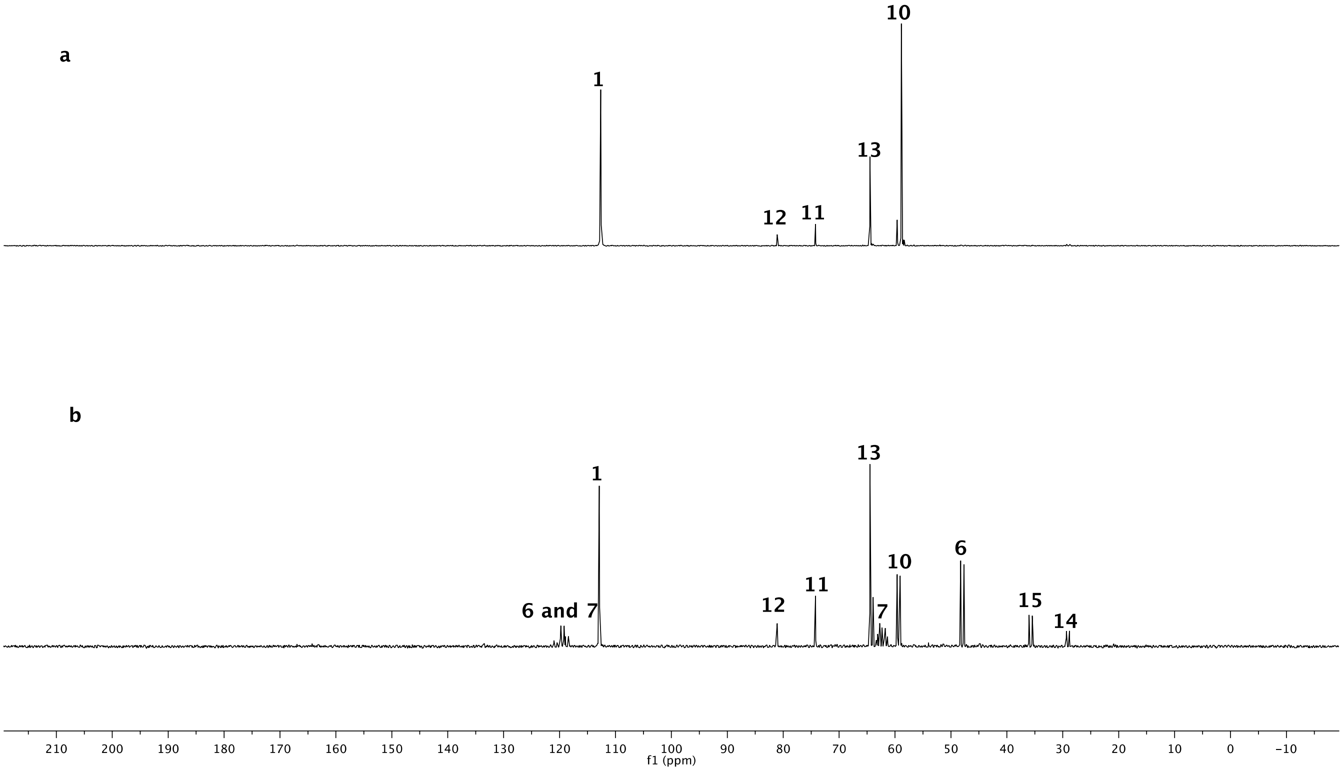

Photoreduction of the mixture of 240 mM ^{13}C-labelled hydroxymethanesulfonate 11, 240 mM ^{13}C-labelled KCN and 240 mM NaH_2PO_4 at pH 7. **a)** ^{13}C NMR spectrum of the mixture after 2.5 h irradiation; **b)** ^{13}C NMR spectrum of the mixture after 5 h irradiation.

4.2.2 ^{13}C NMR spectra of the spiking experiments for the photoreduction of ^{13}C-labelled KCN with sodium sulfite

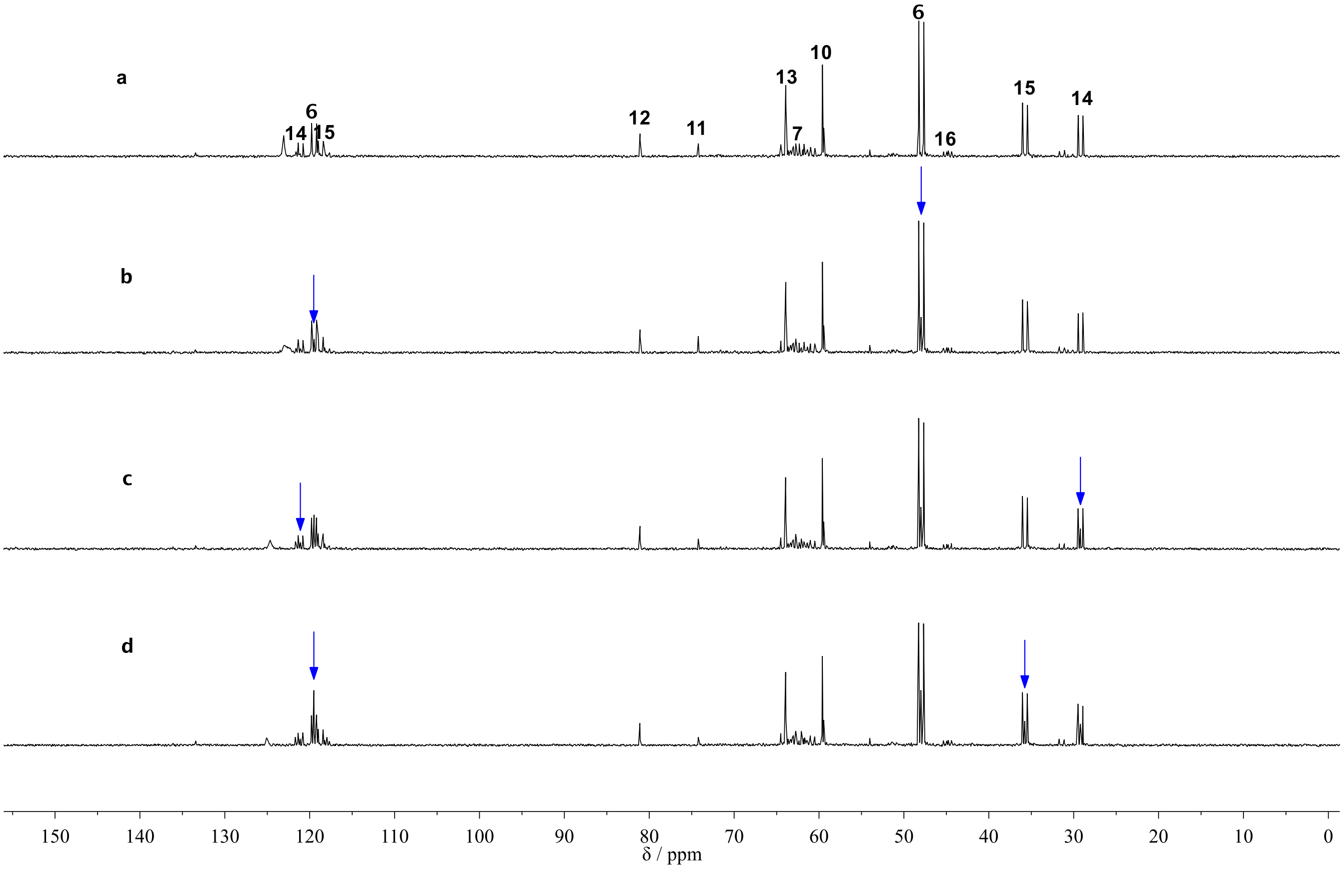

a) ^{13}C NMR spectrum of reaction mixture at pH 9.2 overnight after irradiation for 5 h; **b)** as a), spiked with unlabelled glycolonitrile **6**; **c)** as b), spiked with unlabelled aminoacetonitrile **14**; **d)** as c), spiked with unlabelled iminodiacetonitrile **15** Signals from the spiking compounds are indicated with blue arrows, as shown above.

4.2.3 ^{13}C NMR spectra of the spiking experiments for the photoreduction of ^{13}C-labelled hydroxymethanesulfonate (11) with ^{13}C-labelled KCN

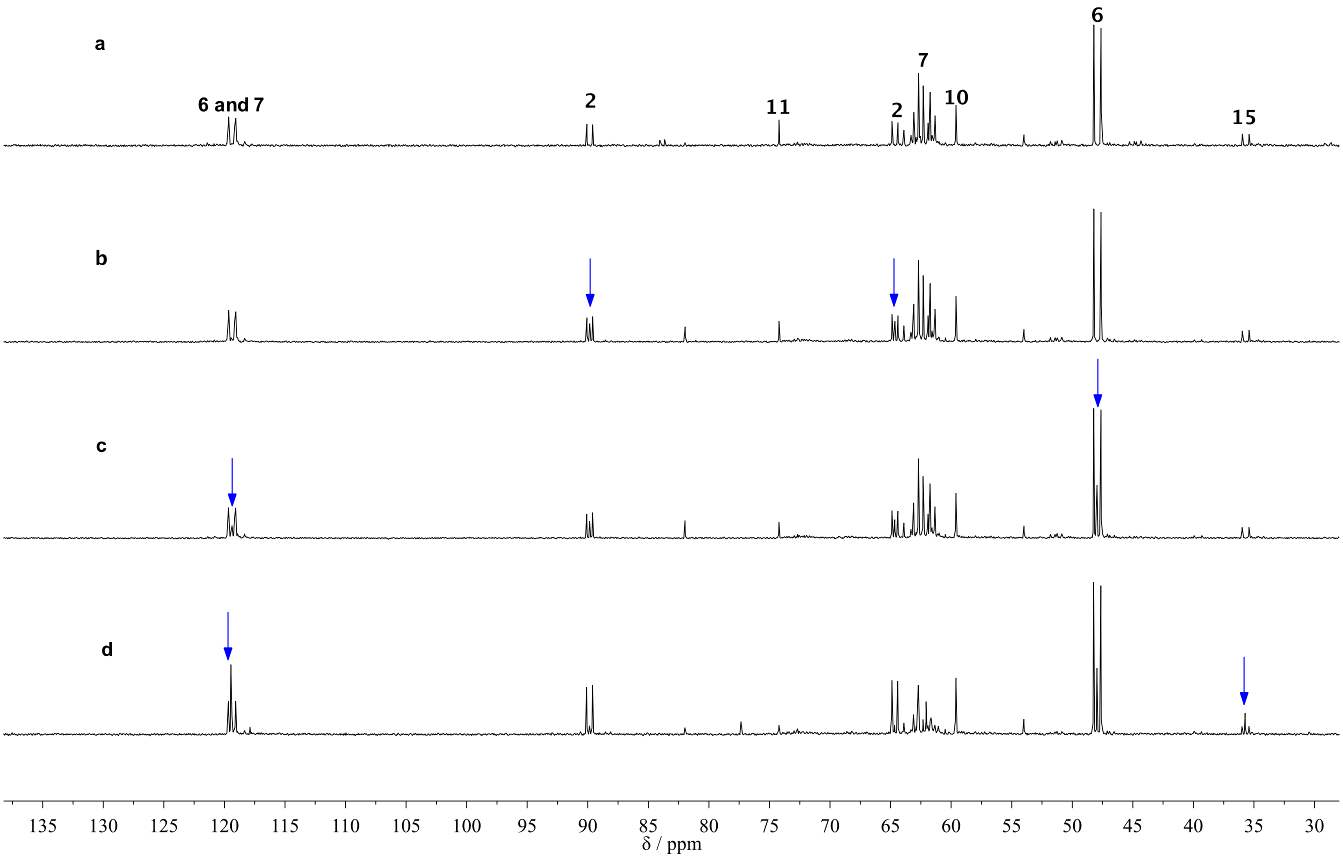

a) ^{13}C NMR spectrum of reaction mixture after irradiation for 12.5 h and sparging with argon; **b)** as a), spiked with unlabelled glycoaldehyde **2**; **c)** as b), spiked with unlabelled glycolonitrile **6**; **d)** as c), spiked with unlabelled iminodiacetonitrile **15**. Signals from the spiking compounds are indicated with blue arrows, as shown above.

4.2.4 Quantitative ^{13}C NMR spectrum of the photoreduction of ^{13}C-labelled hydroxymethanesulfonate (11) with ^{13}C-labelled KCN

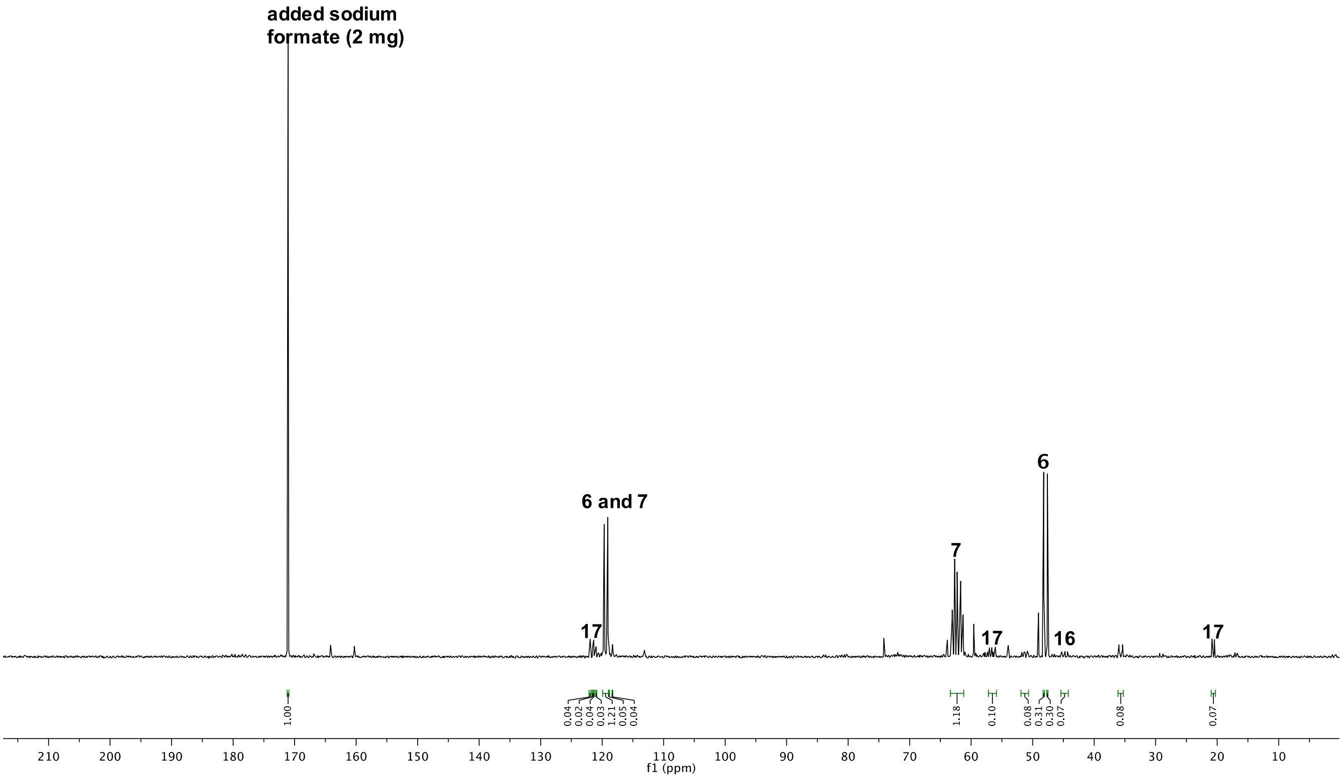

Quantitative ^{13}C NMR spectrum of reaction mixture before purging argon with added ^{13}C-labelled sodium formate (2 mg) as internal reference.

4.2.5 ^{13}C NMR spectra of the photoreductions of ^{13}C-labelled hydroxymethanesulfonate (11) with ^{13}C-labelled KCN with or without ferrocyanide

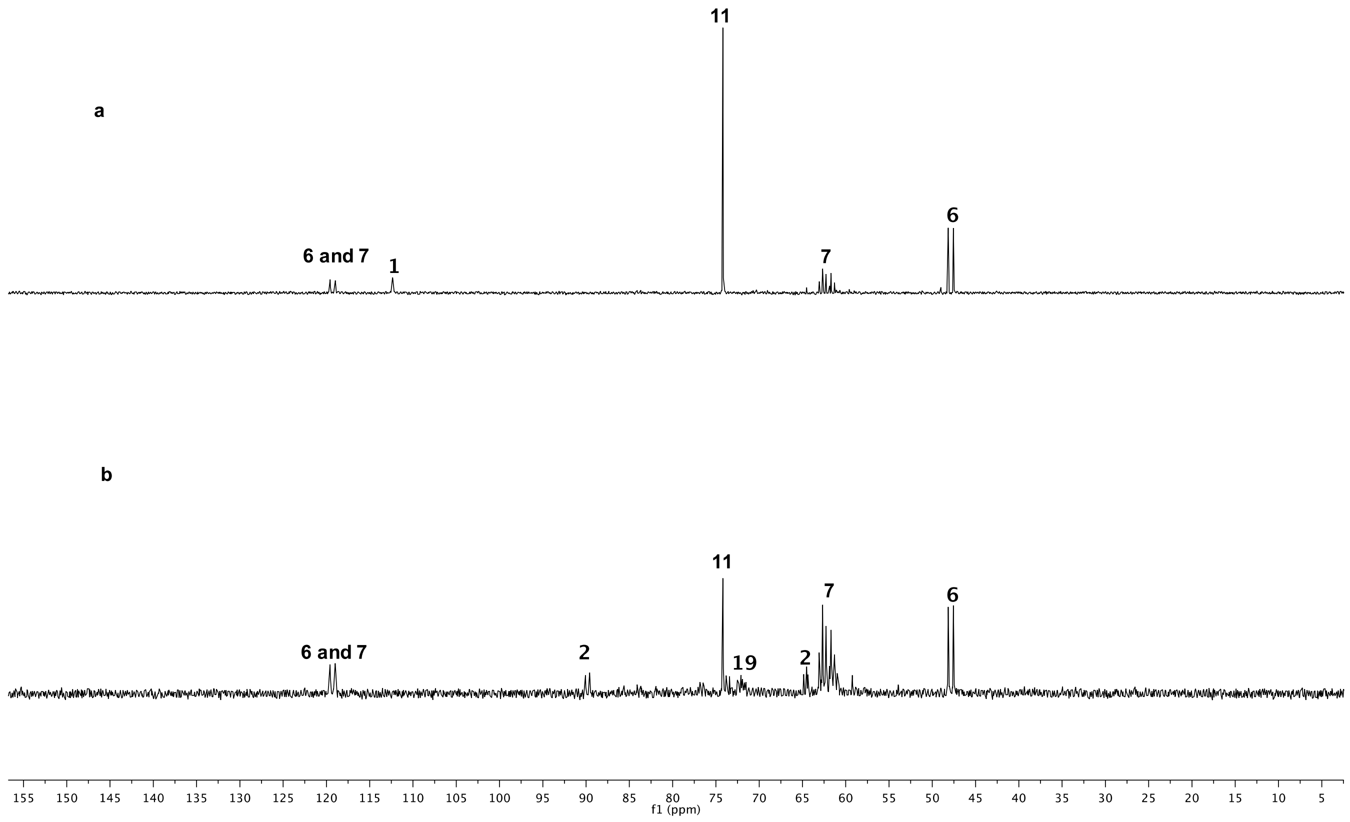

Photoreduction of the mixture of 25 mM ^{13}C-labelled hydroxymethanesulfonate **11**, 25 mM ^{13}C-labelled KCN and 100 mM NaH_2PO_4 at pH 7. **a)** ^{13}C NMR of the mixture with 10 mol% ferrocyanide after 3 h irradiation; **b)** ^{13}C NMR of the mixture without ferrocyanide after 3 h irradiation.

4.2.6 ^{1}H NMR time course of the photoreductions of hydroxymethanesulfonate (11) with KCN with or without ferrocyanide

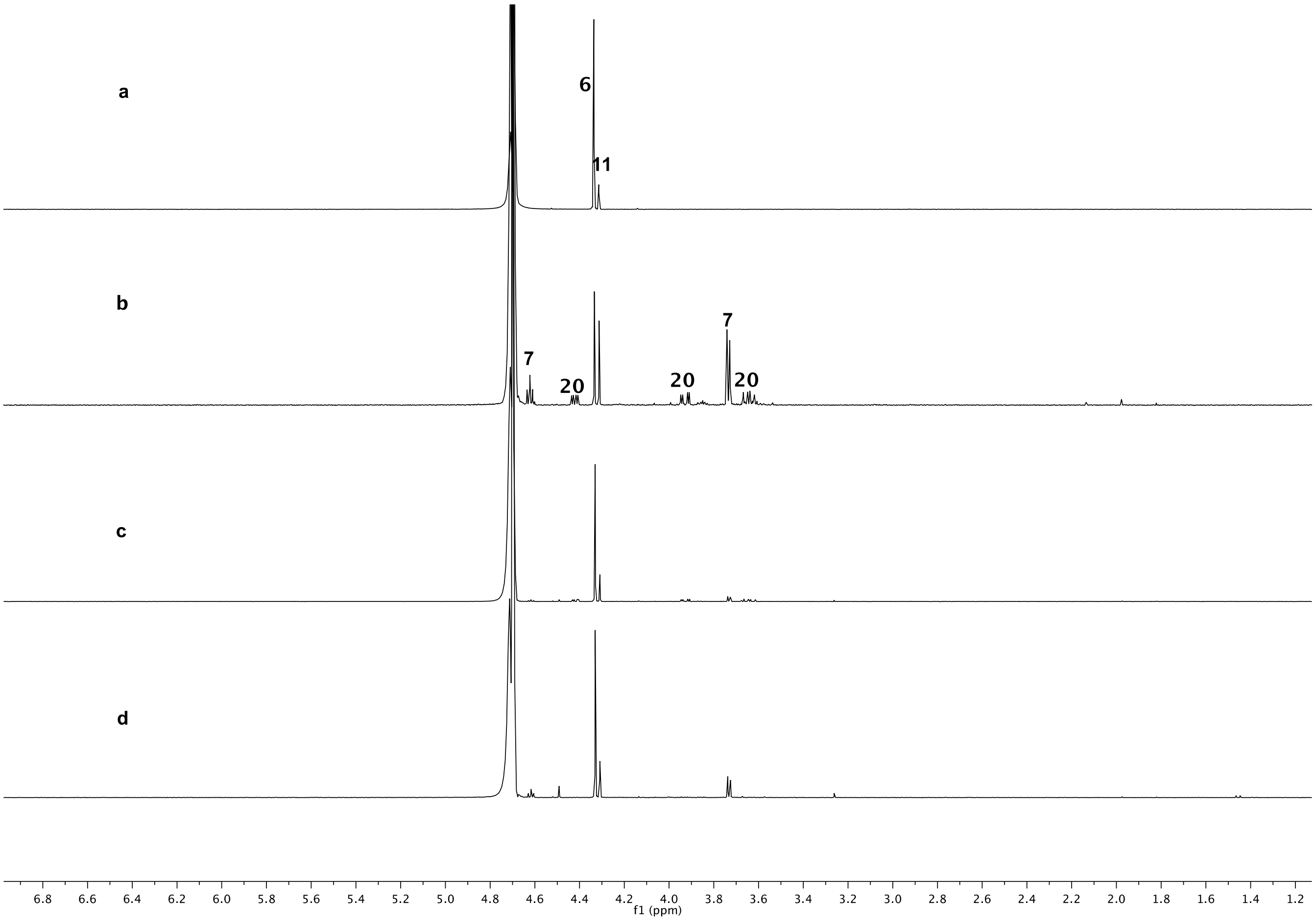

Photoreduction of the mixture of 25 mM hydroxymethanesulfonate **11**, 25 mM KCN and 100 mM NaH_2PO_4 at pH 7. **a)** ^{1}H NMR of the mixture in the dark; **b)** ^{1}H NMR of the mixture after 1 h irradiation with 10 mol% $K_4Fe(CN)_6$; **c)** ^{1}H NMR of the mixture after 1 h irradiation without ferrocyanide; **d)** ^{1}H NMR of the mixture after 3 h irradiation without ferrocyanide.

4.2.7 ^{1}H NMR time course of the photoreduction of 1,2-dihydroxypropanenitrile (7) with sodium sulfite with or without ferrocyanide

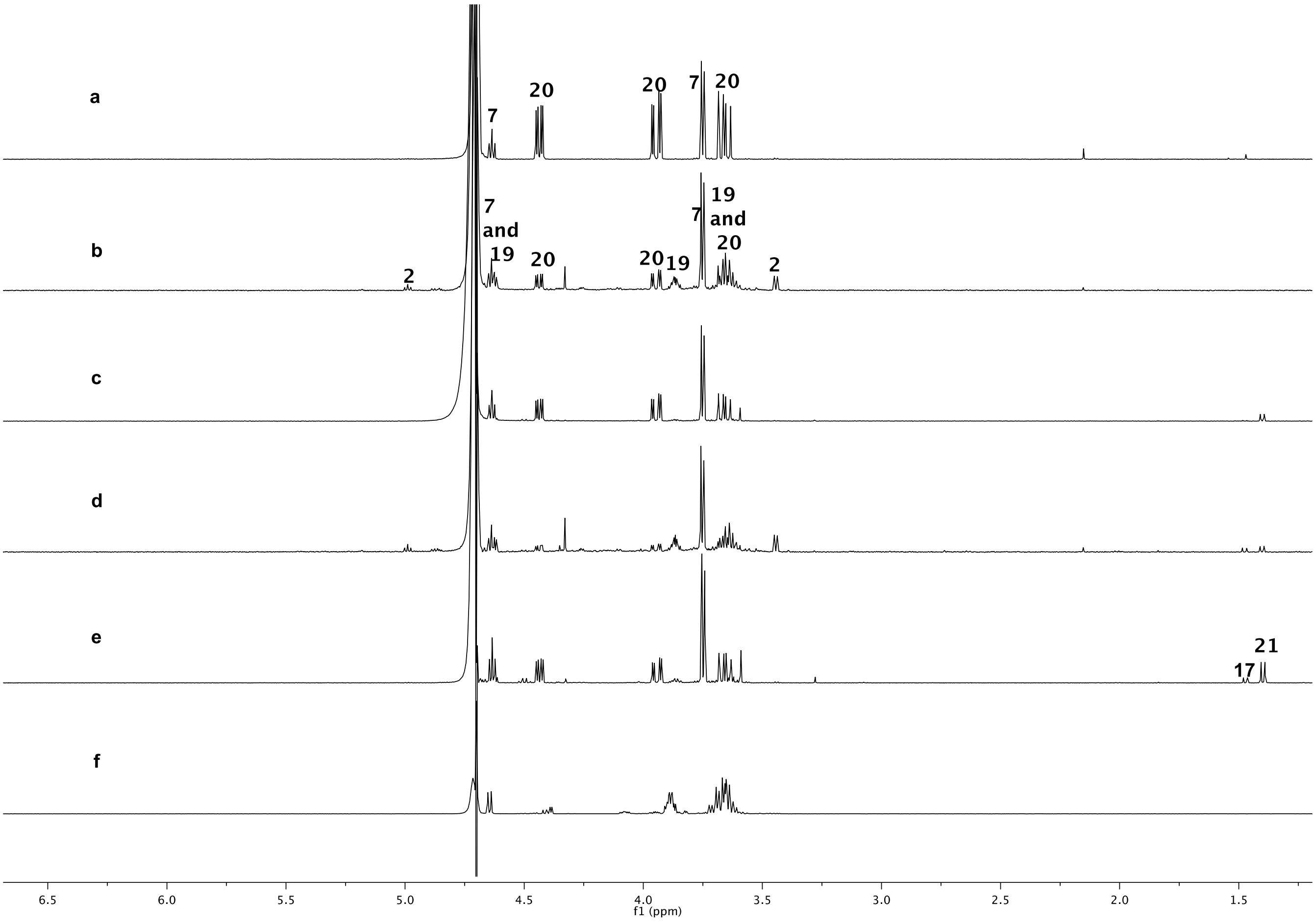

Photoreduction of the mixture of 25 mM glycoaldehyde **2**, 25 mM KCN, 25 mM Na_2SO_3 and 100 mM NaH_2PO_4 at pH 7. **a)** ^{1}H NMR of the mixture in the dark; **b)** ^{1}H NMR of the mixture after 1 h irradiation with 10 mol% $K_4Fe(CN)_6$; **c)** ^{1}H NMR of the mixture after 1 h irradiation without ferrocyanide; **d)** ^{1}H NMR of the mixture after 3 h irradiation with 10 mol% $K_4Fe(CN)_6$; **e)** ^{1}H NMR of the mixture after 3 h irradiation without ferrocyanide; **f)** ^{1}H NMR of 2,3,4-trihydroxybutanenitrile **19** (prepared by mixing glyceraldehyde **3** with KCN).

References

[1] D. Ritson and J. D. Sutherland, *Nat. Chem.*, 2012, **4**, 895–899.
[2] D. J. Ritson and J. D. Sutherland, *Angew. Chem. Int. Ed.,* 2013, **52**, 5845–5847.
[3] M. W. Powner, B. Gerland and J. D. Sutherland, *Nature*, 2009, **459**, 239–242.
[4] B. H. Patel, C. Percivalle, D. J. Ritson, C. D. Duffy and J. D. Sutherland, *Nat. Chem.*, 2015, **7**, 301–307.
[5] J. Xu, M. Tsanakopoulou, C. J. Magnani, R. Szabla, J. E. Šponer, J. Šponer, R. W. Góra and J. D. Sutherland, *Nat. Chem.*, 2017, **9**, 303–309.
[6] S. Ranjan and D. D. Sasselov, *Astrobiology,* 2016, **16**, 68–88.
[7] G. M. Marion, J. S. Kargel, J. K. Crowley and D. C. Catling, *Icarus,* 2013, **225**, 342–351.
[8] I. Halevy, M. T. Zuber and D. P. Schrag, *Science,* 2007, **318**, 1903–1907.
[9] L. Kaltenegger and D. D. Sasselov, *Astrophys. J.*, 2010, **708**, 1162–1167.
[10] E. Hayon, A. Treinin and J. Wilf, *J. Am. Chem. Soc.,* 1972, **94**, 47–57.
[11] Om P. Chawla, N. L. Arthur and R. W. Fessenden, *J. Phys. Chem.,* 1973, **77**, 772–776.
[12] A. S. Jeevarajan and R. W. Fessenden, *J. Phys. Chem.,* 1989, **93**, 3511–3514.

[13] A. D. Keefe and S. L. Miller, *Orig. Life Evol. Biosph.,* 1996, **26**, 111–129.
[14] M. Shirom and G. Stein, *J. Chem. Phys.,* 1971, **55***,* 3379–3382.
[15] M. Reinhard, G. Auböck, N. A. Besley, I. P. Clark, G. M. Greetham, M. W. D. Hanson-Heine, R. Horvath, T. S. Murphy, T. J. Penfold, M. Towrie, M. W. George and M. Chergui, *J. Am. Chem. Soc.,* 2017**, 139**, 7335–7347.
[16] G. B. Seifer, *Russ. J. Inorg. Chem*., 1962, **7**, 640–643.
[17] M. Shirom and G. Stein, *J. Chem. Phys.,* 1971, **55***,* 3372–3378.
[18] R. S. Murray, *Chem. Commun.,* 1968, 824–825.
[19] J. M. Lancaster and R. S. Murray, *J. Chem. Soc. A*, 1971, 2755–2758.
[20] A. D. James and R. S. Murray, *Inorg. Nucl. Chem. Lett*., 1976, **12**, 739-742.
[21] R. A. B. Bannard, J. H. Ross, *Can. J. Chem.* **1954**, *32*, 49-50.

Author contributions

All the authors conceived the project. J. X. & D. J. R. carried out the experiments guided by the geochemical & atmospheric chemistry considerations of S. R., Z. R. T. & D. D. S. and supervised by J. D. S. who co-wrote the paper with J. X.
